# Factors influencing health service utilization among mothers for under-five children: A cross-sectional study in Khulna district of Bangladesh

**DOI:** 10.1371/journal.pone.0274449

**Published:** 2022-09-12

**Authors:** Shahinur Akter

**Affiliations:** Sociology Discipline, Social Science School, Khulna University, Khulna, Bangladesh; Bangladesh Agricultural University, BANGLADESH

## Abstract

Although Bangladesh has made significant progress in reducing child mortality, proper utilization of health services for under-five children among mothers remains one of the major challenges. Hence, this study was designed to investigate the factors influencing health service utilization among mothers for under-five children in the Khulna district of Bangladesh. Administering a semi-structured interview schedule, data were collected from 364 randomly selected mothers from the study area between June and August 2021. At first, Pearson’s Chi-square test was conducted to measure the association between outcome and predictor variables. Multivariable logistic regression model was used to identify the factors associated with utilization of health services. Overall, about 59 percent of the mother received health services from unqualified doctors during their children’s illness and the rest of them (41.5%) seek care from the qualified doctors. Results of regression analysis revealed that long duration of illness (AOR = 2.338; CI: 1.175–4.649; *p* = 0.015), the severity of illness (AOR = 6.402; CI: 3.275–12.513; *p*<0.001), and higher cost of treatment (AOR = 7.371; CI: 3.297–16.480; *p*<0.001) were the significant predictors of utilization of health services from the qualified doctors for under-five children. Thus, the study suggests that to reduce under-five child mortality by ensuring proper utilization of health services, it is necessary to raise awareness among mothers, improve transport facilities, establish need-based health care centers, and lower treatment costs.

## Introduction

Infant and child mortality continue to be a major public health problem in the world as well as in Bangladesh [1]. Despite the existence of national programs for improving child health in Bangladesh, child morbidity and mortality continue to be high. The utilization of child healthcare services is poor in rural areas, causing a significant impact on health which increased morbidity and mortality [2]. It is a very concerning issue that approximately 7.4 million children and adolescents died in 2019, mostly from preventable or treatable causes, with 70 percent (5.2 million) being under the age of five, and current trends predict that nearly 48 million under the age of five will die between 2020 and 2030 [3]. Furthermore, in a worldwide context, newborn mortality accounts for 44 percent of under-five mortality and nearly 60 percent of newborn mortality in South Asia [4].

Bangladesh has made significant progress in reducing the under-five child mortality rate during the last two decades. Despite achieving Millennium Development Goal (MDG) 4 by reducing child mortality, Bangladesh still ranks 53rd with the highest child mortality rate which accounts for up to 30.8 percent of the total [5]. Pneumonia, diarrhea, respiratory diseases, injury, and malnutrition were the leading causes of death of under-five children in Bangladesh [6]. Care-seeking interventions have the potential to reduce child mortality, but numerous children in developing countries die because the illness was not recognized in time and treated appropriately by trained medical personnel [7, 8].

In the context of developing countries, mothers are the main caregivers of the children, and hence their healthcare-seeking behavior is crucial to reducing child mortality in Bangladesh [9]. The utilization of healthcare services is a multifaceted behavioral phenomenon. Empirical studies have found that the utilization of healthcare services was influenced by different factors such as age, occupation, education, financial condition, knowledge, family size, previous experience with child deaths, and so on [10–12]. Lack of knowledge about the severity of the disease makes the mothers delay in seeking treatment that causes unexpected deaths of children [10]. Most of the mother in developing country practice self-medication due to a lack of knowledge which makes the medical treatment less fruitful and unexpected death of the child [13].

Availability of healthcare services determine the decisions of seeking healthcare with socio-economic factors like religious and cultural norms, cost of seeking health care, and acceptability of treatment practices [14]. For some the common childhood illnesses, individual socio-cultural factors play an important role in terms of deciding to seek health treatment [15]. Knowledge gaps on the severity of the disease makes the mothers delay in seeking treatment of healthcare that causing unexpected child death [10]. Demographic, socioeconomic, and environmental factors were reported to be associated with healthcare-seeking behavior of under-five children [16]. In developing countries, the major influencing factors of seeking healthcare for children were service cost, distance to the nearest health center, discontent with the quality of care, lack of transportation facilities, and excessive cost of healthcare services [17].

The use of child health services is an effective means of reducing the risk of child morbidity and mortality, especially in places where child health services utilization is poor [18]. In addition, analyzing the factors influencing the utilization of health services can be useful for taking appropriate decisions and policies [19]. However, few studies were conducted in Bangladesh about the healthcare-seeking behavior of mothers for childhood illnesses. Therefore, the present study aims to analyze the factors influencing health service utilization among mothers of under-five children in the Khulna district of Bangladesh.

## Theoretical framework

The behavioral model of health services use is the most frequently cited model of healthcare service utilization [20] and has been used to guide the examination of predictors associated with various outcomes. This model was developed by the US medical sociologist and health services researcher Ronald M. Andersen in 1968 [21]. This model emphasizes on three core factors to explain healthcare utilization: predisposing factors, enabling factors, and need factors (Andersen, 2008). Andersen & Davidson described these three major components in their most recent explication of the model [22]. Predisposing factors include demographic characteristics such as age and sex, social factors including education, occupation, ethnicity as well as social relationships (e.g., family status), and mental factors in terms of health beliefs (e.g., attitudes, values, and knowledge related to health and health services). Enabling factors consist of income and wealth of an individual, cost of treatment, means of transportation, travel time, and waiting time for healthcare. It also includes the amount, varieties, locations, structures and distribution of health services facilities, and personnel. Need factors to focus on the perceived need for health services (i.e., how people view and experience their general health, functional state, and illness symptoms) and evaluated need (i.e., professional assessments and objective measurements of patients’ health status and need for medical care).

**Fig 1:**
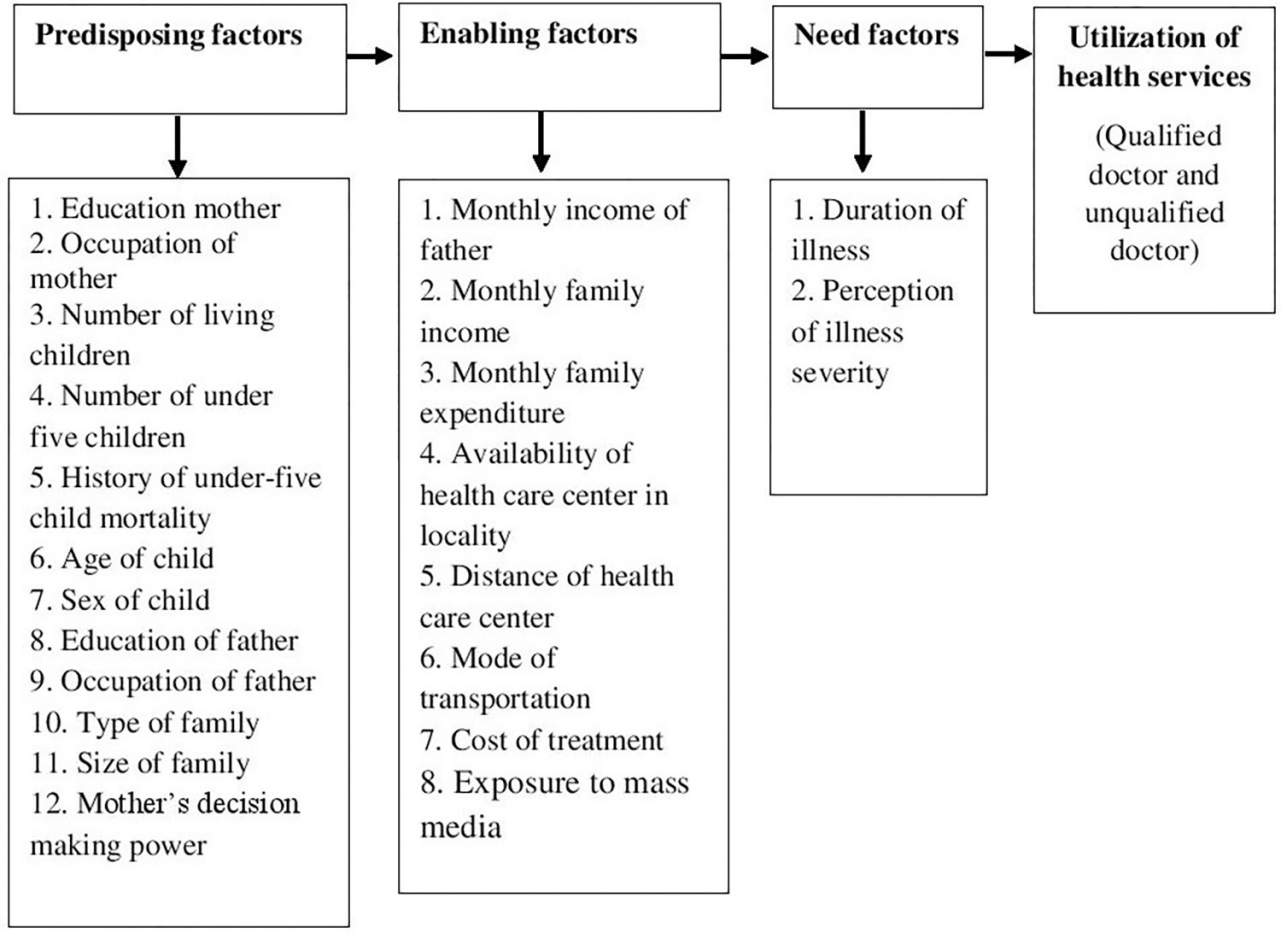
Conceptual framework based on Andersen’s behavioral model.

This model has recently been applied in numerous systematic reviews on different aspects of healthcare utilization to structure their results [23–26]. The behavioral model has been applied in the present study to assess the factors influencing the utilization of healthcare services for under-five children (Fig 1). Different factors were considered under three main components e.g. predisposing, enabling, and need factors of the behavioral model. Within predisposing factors, variables such as education of mother, occupation of mother, number of living children, number of under-five children, history of under-five child mortality, age of the child, sex of the child, education of father, occupation of father, type of family, size of family, and mother’s decision making power were considered. Moreover, enabling factors include the monthly income of the father, monthly family income, monthly family expenditure, availability of the healthcare center in the locality, distance of the healthcare center, mode of transportation, cost of treatment, and exposure to mass media. Besides, need factors to include duration of illness and severity of illness.

## Materials and methods

### Study area

This study had been conducted at two villages namely *Raingamari* village of Batiaghata Upazila and *Pankhali* village under Dacope Upazila in Khulna District of Bangladesh. This area had been purposively selected to explain the accessibility and availability of health services for children in rural areas of Bangladesh as well as to identify the factors influencing health service utilization among mothers for under-five children. Furthermore, research on factors influencing health service utilization among mothers for their children was not conducted particularly, amongst younger age groups in this area.

### Sampling

As mothers are the primary caregiver in Bangladesh, mothers having at least one under-five children and residing in the study area with their spouse were considered as the unit of analysis. A census with trained personnel had been conducted during May 2021 in the study area to get information about the population size considering the unit of analysis. During the census, information particularly, the name and age of the children and their parents’ name have been collected. After conducting the census, a population list has been prepared with the above-mentioned information and a serial number has been given. The population size of the selected study area was 502 mothers by the conducted census. Furthermore, a sample of 364 mothers had been calculated for the survey following the simple random sampling technique with a 95 confidence level and a confidence interval of 2.7. For conducting the field survey, mothers had been randomly selected following the lottery method, and final data were collected with the replacement method.

### Data collection and processing

A semi-structured interview schedule was used for data collection. A pre-test on 10 mothers (5 mothers from each village) had been conducted with trained personnel in May 2021 to verify the consistency and accuracy of the data collection tool. Final survey data were collected from 364 participants between June and August 2021 from the study area. However, the respondents who participated in the pre-test opted out of the final data collection process.

Dependent and independent variables were incorporated in this study. The dependent variable in this study was the utilization of health services which was dichotomized into 1 = qualified doctor (doctor of govt./private hospital) and 0 = unqualified doctor (*kabiraj*/self-medicine/homeopathic/paramedic/pharmacy). Different demographic, socioeconomic, and health-related factors were considered as independent variables under three heading as predisposing, enabling, and need factors in this study.

Within predisposing factors, variables like education of mother (non-literate [0], primary [1–5], secondary [6–12], and tertiary [≥13]), occupation of the mother (housewife and working-mother), number of living children (1 and ≥2), number of under-five children (1 and ≥2), history of under-five child mortality (no and yes), age of the child (<2 years, 2–3 years, and >3 years), sex of the child (girl and boy), education of father (non-literate [0], primary [1–5], secondary [6–12], and tertiary [≥13]), occupation of the father (business, self-employed, day labor and employee), type of family (extended and nuclear), size of family (3–4, 5–6, and ≥7), the mother’s decision-making power. An index was developed to measure mother’s decision making power which included 10 statements and assessed into yes = 1 and no = 0. Then, the score was summed up and the total score was further classified into 3 categories namely low (≤4), medium (5–7), and high (8–10).

Besides, enabling factors to include the monthly income of the father (BDT ≤10000, 10001–20000, and ≥20001), monthly family income (BDT ≤10000, 10001–20000, and ≥20001), monthly family expenditure (BDT ≤8000, 8001–16000, and ≥16001), availability of healthcare center in the locality (no and yes), distance of the healthcare center (≤3km, 3.1-6km, and ≥6.1 km), mode of transportation (walking and vehicles), cost of treatment (BDT ≤500, 501–1000, and ≥1001), and mother’s exposure to mass media (no and yes). On the other hand, need factors to include duration of illness (1–3, 4–6, and ≥7 days), and perception of illness severity (not severe and severe).

### Data analysis

Data were analyzed by using Statistical Package for the Social Sciences (SPSS) version 21. Socioeconomic, demographic, and health-related variables, as well as health care seeing behavior, were analyzed by percentage distribution. Pearson’s Chi-square (χ2) test of independence was conducted to assess the association between outcome and predictor variables considering *p*<0.10 as a cut-off value [27–29]. Multivariable logistic regression model was used to identify the factors associated with utilization of health services. Hosmer and Lemeshow test has been executed for model fit, where the *p*-value is 0.331 which ensures model fitness. No multicollinearity has been detected among the variables as the value of VIF ranges from 1.02 to 1.32 and the mean VIF is 1.15. Results of multivariable logistic regression were presented by adjusted odds ratios (AOR) with 95% confidence intervals (CI).

### Ethical approval

Ethical approval was obtained from the Ethical Clearance Committee of Khulna University, Bangladesh (Reference Number-KUECC-2021/11/39). Data were collected from the participants by seeking verbal consent during the field survey. It was assured that the participants could opt out of the survey at any time without any reason.

## Results

### Background characteristics of the participants

Data presented in Table 1 show that the highest of the mother (60.7%) had a secondary level of education and the lowest portion of them were illiterate (4.1%). About 90 percent of the mother were housewives and only 10.7 percent of them were working-mother. The majority of the mother reportedly has more than or equal to two living children (61.5%) as well as one under-five child (93.7%) respectively. The majority of the mother (86.5%) did not have under-five child mortality history and the rest of them (13.5%) experienced under-five child mortality. Regarding the youngest child of the family, the majority of the children were between 2 to 3 years and more than half of them (53%) were girl children. Among the father, a majority had secondary education (39%) and were self-employed (44.5%) as well as monthly earned BDT ≤10,000 (48.9%). More than half of the family’s monthly income was between BDT 10,001 to 20000 (51.4%) and monthly expenditure was between BDT 8001 to 16000 (61.3%). The majority of the respondents were from extended family (52.7%) as well as consisted of 3 to 4 members (42%). The highest of the mother reported that their children suffered from 1 to 3 days (44%), 4 to 6 days (25%), and ≥7 days (31%) respectively during their illness. A nearly three-fourth portion of the mother perceived that the illness was not severe and a majority of the mother mentioned that there was the availability of healthcare centers in their locality. Regarding the distance of the healthcare center, nearly 60 percent of the mother said that the distance of healthcare center was ≤3km. and the rest of them reported the distance was more than 3 km. A significant portion of the mother (65.4%) mentioned that vehicles were their mode of transportation while seeking healthcare. Regarding the cost of treatment, the majority of the mother spent BDT ≤500 during their children’s ailment. About 46 percent of the mother’s decision-making power was reportedly high and nearly three-fourth portion of the mother (74.5%) were exposed to mass media.

**Table 1:** Univariable association between explanatory variable and health service utilization among mothers for under-five children in Khulna district of Bangladesh.

Characteristics		*n* (%)	Health service utilization			Chi-square *p* value
			Unqualified doctor (%)	Qualified doctor (%)		
**Total**		364 (100.0)	213 (58.5%)	151 (41.5%)		
**Predisposing factors**						
**Education of mother**						
	Non-literate	15 (4.1)	9 (60.0)	6 (40.0)		
	Primary	78 (21.4)	50 (64.1)	28 (35.9)		0.626
	Secondary	251 (69.0)	144 (57.4)	107 (42.6)		
	Tertiary	20 (5.5)	10 (50.0)	10 (50.0)		
**Occupation of mother**						
	Housewife	325 (89.3)	196 (60.3)	129 (39.7)		0.058
	Working mother	39 (10.7)	17 (43.6)	22 (56.4)		
**Number of living children**						
	1	140 (38.5)	79 (56.4)	61 (43.6)		0.585
	≥2	224 (61.5)	134 (59.8)	90 (40.2)		
**Number of under-five children**						
	1	341 (93.7)	196 (57.5)	145 (42.5)		0.132
	≥2	23 (6.3)	17 (73.9)	6 (26.1)		
**History of under-five child mortality**						
	No	315 (86.5)	187 (59.4)	128 (40.6)		0.438
	Yes	49 (13.5)	26 (28.7)	23 (20.3)		
**Age of the children**						
	<2 years	88 (24.2)	49 (55.7)	39 (44.3)		
	2–3 years	145 (39.8)	79 (54.5)	66 (45.5)		0.178
	>3 years	131 (36.0)	85 (64.9)	46 (35.1)		
**Sex of the children**						
	Girl	193 (53.0)	112 (58.0)	81 (42.0)		0.915
	Boy	171 (47.0)	101 (59.1)	70 (40.9)		
**Education of father**						
	Non-literate	21 (5.8)	13 (61.9)	8 (38.1)		0.727
	Primary	131 (36.0)	81 (61.8)	50 (38.2)		
	Secondary	178 (48.9)	99 (55.6)	79 (44.4)		
	Tertiary	34 (9.3)	20 (58.8)	14 (41.2)		
**Occupation of father**						
	Business	40 (11.0)	20 (50.0)	20 (50.0)		0.367
	Self-employed	162 (44.5)	102 (63.0)	60 (37.0)		
	Labor	133 (36.5)	76 (57.1)	57 (42.9)		
	Employee	29 (8.0)	15 (51.7)	14 (48.3)		
**Type of family**						
	Extended	192 (52.7)	177 (60.9)	75 (39.1)		0.339
	Nuclear	172 (47.3)	96 (55.8)	76 (44.2)		
**Size of the family**						
	3–4	153 (42.0)	84 (54.9)	69 (45.1)		
	5–6	146 (40.1)	89 (61.0)	57 (39.0)		0.496
	≥7	65 (17.9)	40 (61.5)	25 (38.5)		
**Mother’s decision making power**						
	Low	62 (17.0)	38 (61.3)	24 (38.7)		
	Medium	133 (36.5)	80 (60.2)	53 (39.8)		0.714
	High	169 (46.4)	95 (56.2)	74 (43.8)		
**Enabling Factors**						
**Monthly income of father (in BDT)**						
	≤10000	178 (48.9)	110 (61.8)	68 (38.2)		0.410
	10001–20000	168 (46.2)	94 (56.0)	74 (44.0)		
	≥20001	18 (4.9)	9 (50.0)	9 (50.0)		
**Monthly family income (in BDT)**						
	≤10000	126 (34.6)	84 (66.7)	42 (33.3)		0.070
	10001–20000	187 (51.4)	101 (54.0)	86 (46.0)		
	≥20001	51 (14.0)	28 (54.9)	23 (21.2)		
**Monthly family expenditure (in BDT)**						
	≤8000	65 (17.9)	45 (69.2)	20 (30.8)		0.119
	8001–16000	223 (61.3)	128 (57.4)	95 (42.6)		
	≥16001	76 (20.9)	40 (52.6)	36 (47.4)		
**Availability of healthcare center in locality**						
	No	152 (41.8)	93 (61.2)	59 (38.8)		0.391
	Yes	212 (58.2)	120 (56.6)	92 (43.4)		
**Distance of healthcare center (in km)**						
	≤3	217 (59.6)	130 (59.9)	87 (40.1)		0.001
	3.1–6	82 (22.5)	57 (69.5)	25 (30.5)		
	>6	65 (17.9)	26 (40.0)	39 (60.0)		
**Mode of transportation**						
	Walking	126 (34.6)	75 (59.5)	51 (40.5)		0.823
	Vehicles	238 (65.4)	138 (58.0)	100 (42.0)		
**Cost of treatment (in BDT)**						
	≤500	239 (65.7)	183 (76.6)	56 (23.4)		
	501–1000	68 (18.7)	18 (26.5)	50 (73.5)		<0.001
	≥1001	57 (15.7)	12 (21.1)	45 (78.9)		
**Exposure to mass media**						
	No	93 (25.5)	58 (62.4)	35 (37.6)		0.396
	Yes	271 (74.5)	155 (57.2)	116 (42.8)		
**Need factors**						
**Duration of illness (in days)**						
	1–3	160 (44.0)	126 (78.8)	34 (21.3)		
	4–6	91 (25.0)	46 (50.5)	45 (495)		<0.001
	≥7	113 (31.0)	41 (36.3)	72 (63.7)		
**Perception of illness severity**						
	Not-severe	264 (72.5)	192 (72.7)	72 (27.3)		<0.001
	Severe	100 (27.5)	21 (21.0)	79 (79.0)		

### Factors associated with utilization of health services

A Chi-square test was conducted to assess the relation of different socioeconomic, and demographic factors to the utilization of health services among mothers of under-five children (Table 1). Findings of univariable analysis revealed that among the independent variables, occupation of mother, monthly family income, duration of illness, perception of illness severity, the distance of healthcare center, and cost of treatment were associated with utilization of health services (qualified doctor/unqualified doctor) at *p*<0.10. These variables were included in the multivariable logistic regression analysis.

Out of 22 variables, 6 were found statistically significant with utilization of health services from the univariable analysis. These variables were included in the multivariable logistic regression analysis (Table 2). Here, utilization of health services (qualified doctor and unqualified doctor) was the outcome variable and the predictors were the mother’s occupation, monthly family income, the distance of health care center, cost of treatment, duration of illness, and perception of illness severity. Results reveal that cost of treatment, duration of illness, and perception of illness severity were significantly associated with utilization of health services for under-five children. Furthermore, higher treatment cost was 7.427 times and 7.371 times more likely to be associated with seeking treatment from the qualified doctor compared to lower treatment cost (AOR = 7.427; 95% CI: 3.674–5.015; *p*<0.001) and (AOR = 7.371; 95% CI: 3.297–16.480; *p*<0.001) respectively. Moreover, mothers were 2.226 times more possible to receive treatment from the qualified doctor when the duration of illness was 4 to 6 days compared to the short duration of ailment (AOR = 2.226; 95% CI: 1.128–4.393; *p* = 0.021). Similarly, when the duration of illness was 7 days or more, mothers were 2.338 times more likely to prefer a qualified doctor for seeking treatment during their children’s illness compared to short duration of illness (AOR = 2.338; 95% CI: 1.175–4.649; *p* = 0.015). However, when mothers perceived illness as severe were 6.402 times more possible to receive treatment from the qualified doctors compared to their counterparts (AOR = 6.402; 95% CI: 3.275–12.513; *p*<0.001). On the contrary, occupation of the mother, monthly family income, and distance of healthcare center were not significantly associated with utilization of health services among mothers for under-five children.

**Table 2:** Factors associated with health service utilization in Khulna district of Bangladesh.

Factors		Standardized coefficient			
		β	*p* value	AOR	95% CI (Lower-Upper)
**Enabling factor**					
**Cost of treatment (in BDT)**					
	≤500^(Ref)^				
	501–1000	2.005	<0.001	7.427	3.674–5.015
	≥1001	1.998	<0.001	7.371	3.297–16.480
**Need factors**					
**Duration of illness (in days)**					
	1-3^(Ref)^				
	4–6	0.800	0.021	2.226	1.128–4.393
	≥7	0.849	0.015	2.338	1.175–4.649
**Perception about illness severity**					
	Not-severe^(Ref)^				
	Severe	1.857	<0.001	6.402	3.275–12.513

Ref. Reference group; AOR: Adjusted Odd Ratio; CI: Confidence interval.

## Discussion

Despite worldwide progress in reducing child mortality, a significant number of children died every year due to a lack of proper utilization of health services. Proper utilization of health services may reduce under-five child morbidity and mortality. Therefore, this study aimed to identify the factors influencing health service utilization among mothers of under-five children in the Khulna district of Bangladesh. Findings of the study revealed that about 59 percent of the mother received health services from unqualified doctors during their children’s ailment and the rest of them (41.5%) seek care from the qualified doctors. Furthermore, results of regression analysis exposed that different factors such as cost of treatment, duration of illness, and perception of illness severity were significantly associated with utilization of health services among mothers of under-five children. Andersen’s behavioral model was followed in this study to examine the association between dependent and independent variables.

### Predisposing factors

Mother’s employment status has a significant effect on the utilization of health services for their under-five children during the ailment. It is evident from prior studies [11, 30–33] that working mothers were more possible to receive health services for under-five children. The possible reason could be the working mother might be able to pay for treatment for their children when they bring them to the doctor, something that non-working mothers may be unable to accomplish on their own. However, the findings of the current study indicate that working mother was more likely to receive treatment from qualified doctor compared to mothers who were housewives but this variable was not significantly associated with the utilization of healthcare services which is consistent with some previous studies [34, 35]. This phenomenon might be explained by the fact that the percentage of working-mother in the study area was comparatively low and there might be the influence of other mediating social factors which need further investigation.

### Enabling factors

It is evident from the findings of previous studies in different settings that children from the richest household had a higher likelihood of receiving treatment compared to children from the poorest households [36–41]. This finding could be explained by the fact that mothers who encounter financial difficulties may be unable to pay for healthcare, preventing them from seeking treatment for their children. However, the present study did not find any significant relationship between monthly family income and health service utilization. On the contrary, cost of treatment has a significant association with the utilization of health services for under-five children. Higher treatment cost was more likely to be associated with seeking treatment from the qualified doctors compared to lower treatment cost. Some previous studies [9, 42] also noted that low price was the most powerful issue for visiting drug sellers regularly, and the high cost of medical doctors was the most influential factor for not visiting drug sellers.

### Need factors

Under need factors, duration of illness, and perception of illness severity were significantly associated with utilization of health services for under-five children. Mothers were more possible to receive treatment from the qualified doctors for a long duration of illness of their children (7 days or more) compared to the short duration of illness. It is consistent with the findings of previous studies [9, 43] that found longer sickness persistence was important interpreters for seeking treatment from qualified doctors. Besides, when mothers perceived illness as severe were more possible to receive treatment from the qualified doctors compared to their counterparts. It is aligned with the findings of another study conducted in Bangladesh that revealed that perceived disease severity was found to be important predictor for seeking treatment from qualified doctors [9]. The possible reason for this finding could be the mother’s perception of a longer duration of illness as severe so they prefer proper diagnosis and treatment of their child’s ailment. Similarly, a shorter duration of childhood illness might seem to be not serious and cured automatically or with receiving treatment from the traditional or unqualified doctor.

### Strength and limitations

The major strength of this study is that a simple random sampling technique was followed for selecting participants which is more scientific. In addition, the study followed Andersen’s behavioral model which is a widely accepted method for assessment of factors related to health services utilization. Besides, this study considered a wide array of demographic, socioeconomic, and health-related factors to determine the utilization of health services among mothers of under-five children in the study area. However, this study has some limitations. Firstly, this study was carried out in a specific region which may limit the interpretation of the findings at the national level. Secondly, small sample size may limit the generalizability of the findings for the whole younger age group in Bangladesh. Thirdly, the mother’s response regarding the child’s illness before the study was based on recall which may exhibits recall bias and sometimes lead to underreporting of the child ailment.

## Conclusion

Proper utilization of health services is an important indicator for ensuring better health for children. The study aimed to investigate the factors influencing health service utilization among mothers of under-five children in the Khulna district of Bangladesh. Findings from the multivariable analysis revealed that cost of treatment, duration of illness, and perception of illness severity were significantly associated with utilization of healthcare services among mothers of under-five children. The government of Bangladesh by collaborating with NGOs should strengthen the healthcare system by reducing the financial barriers to accessing health services for under-five children. Furthermore, the government should create favorable conditions to improve the overall socio-economic condition of rural people particularly, rural mothers by fostering education and employment so that they can access better healthcare for their children. Besides, an improvement in rural transport, facility-based healthcare centers, and reduction of treatment costs may increase the possibility of receiving healthcare services from the qualified doctors for under-five children.
